# Aberrations of Genomic Imprinting in Glioblastoma Formation

**DOI:** 10.3389/fonc.2021.630482

**Published:** 2021-03-12

**Authors:** Anna Lozano-Ureña, Esteban Jiménez-Villalba, Alejandro Pinedo-Serrano, Antonio Jordán-Pla, Martina Kirstein, Sacri R. Ferrón

**Affiliations:** ^1^Instituto de Biotecnología y Biomedicina (BIOTECMED), Valencia, Spain; ^2^Departamento de Biología Celular, Universidad de Valencia, Valencia, Spain

**Keywords:** genomic imprinting, glioblastoma, neural stem cells, methylation, subventricular zone

## Abstract

In human glioblastoma (GBM), the presence of a small population of cells with stem cell characteristics, the glioma stem cells (GSCs), has been described. These cells have GBM potential and are responsible for the origin of the tumors. However, whether GSCs originate from normal neural stem cells (NSCs) as a consequence of genetic and epigenetic changes and/or dedifferentiation from somatic cells remains to be investigated. Genomic imprinting is an epigenetic marking process that causes genes to be expressed depending on their parental origin. The dysregulation of the imprinting pattern or the loss of genomic imprinting (LOI) have been described in different tumors including GBM, being one of the earliest and most common events that occurs in human cancers. Here we have gathered the current knowledge of the role of imprinted genes in normal NSCs function and how the imprinting process is altered in human GBM. We also review the changes at particular imprinted loci that might be involved in the development of the tumor. Understanding the mechanistic similarities in the regulation of genomic imprinting between normal NSCs and GBM cells will be helpful to identify molecular players that might be involved in the development of human GBM.

## Genomic Imprinting and Gene Dosage Control

**Figure 1:**
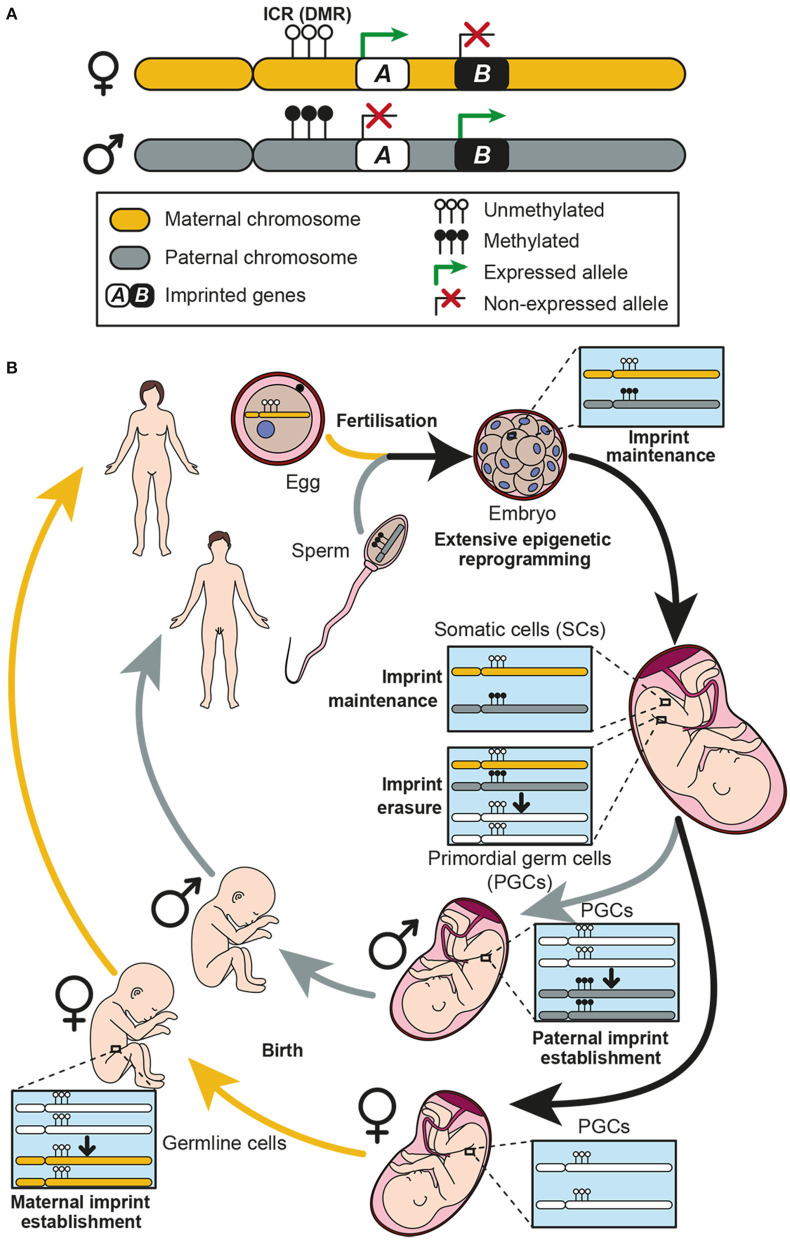
Genomic imprinting and the establishment of imprints in the germline. **(A)** Two homolog chromosomes are represented, each one inherited from one progenitor: maternal chromosome in yellow and paternal chromosome in gray. An imprinting cluster containing two imprinted genes (genes *A* and *B*) is represented. Gene *A* is maternally expressed, while gene *B* is paternally expressed. Expression of both genes is controlled by methylation at the imprinting control region (ICR) which is a differentially methylated region (DMR) between the two chromosomes. **(B)** Genomic imprinting life cycle is represented. When fertilization occurs, the zygote receives a maternal and a paternal copy of the genome, each one imprinted accordingly. Methylation patterns of each chromosome must be kept in somatic cells, thus imprints are protected against the extensive genome demethylation that occurs after fertilization. Imprints are maintained along the individual life in somatic cells, while they are erased in primordial germ cells (PGCs) during development. Afterwards, a new imprint is established in the germline according to the individual chromosomal sex. These imprints are established during development in males and postnatally in females.

Genomic imprinting is an epigenetic process in which a small group of genes, called imprinted genes, are expressed depending on their parental origin (1–3). Whereas non-imprinted genes express both copies contained on homolog chromosomes, in imprinted genes either the maternal or paternal copy is expressed thus bypassing mendelian inheritance laws (4, 5) (Figure 1A). Therefore, parental genomes are not functionally equivalent due to genomic imprinting, implying that both genomes are required for normal mammalian development (6, 7). To date, around 200 imprinted genes have been described in mice (8) and more than 150 in humans (9, 10). Although imprinted genes represent <1% of total genes in the mammalian genome, they play important roles in different biological processes such as embryonic and placenta growth, fetal development and adult metabolism (11–13).

Most imprinted genes are grouped in clusters (3, 14) and it has been postulated that a *cis* regulatory DNA element could regulate the expression of all genes contained in the same cluster (4). Indeed, imprinted gene expression is known to be co-ordinately controlled by epigenetic mechanisms, being DNA methylation the most important one occurring in specific genomic regions enriched in cytosine and guanine dinucleotides (CpG) (15). These regions, known as imprinting control regions (ICRs), are differentially methylated (DMRs) exhibiting a specific parental methylation pattern (2, 14) (Figure 1A). Importantly, deletion of these sequences implies a loss of imprinting (LOI), which results in alterations of the expression of imprinted genes in the cluster (14, 16).

The establishment of imprints takes place in the germline through a multistep mechanism termed imprinting life cycle, which ensures monoallelic expression of imprinted genes (17) (Figure 1B). During embryogenesis, the primordial germ cells (PGCs), which will give rise to the gametes, have the methylation patterns characteristic of somatic cells. However, in the genital ridges, the imprints are erased during gamete formation to allow re-establishment of new parental-specific marks at the ICRs (4, 8). This process takes place during development at different times in males and females (18). Paternal-specific methylation occurs prenatally in pro-spermatogonia before meiosis, whereas maternal-specific ICR methylation takes place postnatally in growing oocytes (19) (Figure 1B). After establishment of imprints, methylation patterns of each chromosome must be kept in somatic cells, thus imprints are protected against the extensive genome demethylation that occurs after fertilization (17), and then transmitted to every somatic cell (10) (Figure 1B).

During development and adult life, genomic imprinting can be modified leading to tissue or cell type specific imprint patterns (2). Indeed, loss of imprinting (LOI) has consequences in physiological processes and is the cause of some human imprinting syndromes such as Angelman, Prader-Willi or Beckwith-Wiedemann, which course with severe neurological defects (3, 9, 20). Moreover, disruption of imprinting can cause a predisposition to tumor formation, and LOI in several genes is considered to be the most common and early event in human cancers such as colorectal or esophageal cancer, meningiomas, gliomas, and chronic myeloid leukemia among others (13, 21–23).

## Imprinted Genes and NSCs

In the mammalian brain, two regions generate new neurons throughout adulthood: the subventricular zone (SVZ) in the walls of the lateral ventricles, and the subgranular zone (SGZ) in the dentate gyrus (DG) of the hippocampus (24, 25). The process of neurogenesis in these adult neurogenic niches is continually sustained by the activity of NSCs, which are characterized by their ability to balance self-renewal with multipotential differentiation into astrocytes, oligodendrocytes and neurons (26). Activated and quiescent NSCs (also known as type B1 cells) coexist in the adult SVZ (27) and once activated, slowly dividing NSCs give rise to fast cycling cells called transit-amplifying progenitors (TAP or type C cells). Mash1-positive type C cells in turn generate immature neurons or neuroblasts (type A cells) that migrate tangentially through the rostral migratory stream (RMS) toward the olfactory bulb (OB). These chains of polysialylated neural cell adhesion molecule (PSA-NCAM) positive neuroblasts reach the core of the OB, where they integrate and differentiate into inhibitory interneurons, playing an important role in rodent olfaction (28). Although less frequently, subventricular NSCs are also capable of producing some oligodendroblasts that migrate to the corpus callosum and striatum, where they differentiate into myelinating and non-myelinating oligodendrocytes (29, 30). The human SVZ is also considered as an important pool of neuronal and glial progenitor cells, and this pool has been implicated in injury, neurodegeneration and cancer (31).

In the SVZ, type B1 cells have many features of astrocytes and retain expression of NESTIN or GLAST (astrocyte-specific glutamate aspartate transporters), markers that are also expressed in radial glia cells (RGCs), the NSCs in the developing brain (32, 33). The majority of NSCs in the adult SVZ originate from these RGC cells between embryonic days (E) 13.5 and 15.5 and remain largely quiescent until they become reactivated postnatally (34, 35).

Recent studies on the developing brain and postnatal neurogenic niches raise many intriguing questions concerning the role of genomic imprinting and gene dosage in gliogenesis and neurogenesis, including how imprinted genes operate in concert with signaling cues to contribute to these processes (36). For example, during cortical neurogenesis, radial glia cells express high levels of the paternally-expressed zinc finger protein *Zac1*, which leads to the expression of other imprinted genes such as the maternally-expressed cyclin-dependent kinase inhibitor *Cdkn1c*, known to promote NSC cell cycle arrest and proglial differentiation (37). Interestingly, *Cdkn1c* has been shown to also promote NSCs quiescence in the adult hippocampus, and long-term deletion of the gene leads to NSC exhaustion and impaired neurogenesis in aged mice (35). Moreover, in the embryonic mouse neocortex, the proliferative capacity of cortical progenitors is repressed by paternal expression of *Necdin*, which suppresses neural progenitor proliferation by antagonizing the polycomb protein BMI1 function (38).

Genomic imprinting can be selectively lost or “*switched off* ” in particular cell types or at specific developmental points to activate an allele that is usually repressed by imprinting (36). For example, in the adult SVZ, the insulin-like growth factor 2 (*Igf2*) gene, canonically expressed from the paternally-inherited allele, is biallelically expressed in the choroid plexus and secreted into the cerebrospinal fluid to regulate NSC proliferation (39, 40). IGF2 is also biallelically expressed in the postnatal human and mouse choroid plexus epithelium and leptomeninges, acting as a paracrine factor that regulates NSC homeostasis (39, 41). In contrast, in the SGZ, *Igf2* is expressed in NSCs in an imprinted manner, suggesting that the regulatory decision to imprint or not is an important mechanism of transcriptional dosage control in adult neurogenesis (39). Another example of LOI in the SVZ is the paternally-expressed gene *Delta-like homolog 1* (*Dlk1*), an atypical Notch ligand located on mouse chromosome 12 (human chromosome 14) that plays a relevant dual function to regulate postnatal neurogenesis (42). *Dlk1* is a single gene that encodes for both a secreted factor (expressed by niche astrocytes) and a bound receptor (expressed by NSCs). *Dlk1*, which is a canonically imprinted gene elsewhere in the brain, shows a selective absence of imprinting in these cell types, and biallelic expression of *Dlk1* is required for stem cell maintenance in the SVZ and final neurogenesis in the olfactory bulb (42). In conclusion, genomic imprinting might be reversible and context-dependent and is likely to be essential to control neural stem cell potential and for normal development and tissue regeneration in the adult brain.

## Genomic Imprinting in Human Glioblastoma

In the central nervous system (CNS), as in many other tissues, diverse types of tumors may emerge throughout life. Gliomas arise from glial cells and are the most frequent primary tumors in the brain (43). According to the criteria established by the World Health Organisation (WHO) in 2016, gliomas are classified as grades I to IV based on histology and clinical criteria (44). Grade I tumors are generally benign and frequently curable, whereas malignant glioma are subdivided from the least aggressive grade II to grade IV, which is more proliferative, more necrosis-prone and angiogenic and has a poorer prognosis (45–47). GBM is the most aggressive and frequent grade IV type glioma and despite its low incidence (3.21 cases per 100,000 people), up to 46% of primary malignant brain tumors are GBM (43, 48). Patients diagnosed with GBM survive on the average 15 months and the 5-year-survival rate is only 5.6% (48, 49).

Due to its frequency and lethality, several studies have been carried out in order to characterize different human GBM subtypes based on genome and transcriptome changes. For example, the epidermal growth factor receptor (EGFR) is altered in almost 50% of GBM and represents one of the most promising therapeutic targets (50). Other mutations affecting TP53, PTEN, RB1, ERBB2, PIK3R1 or PIK3CA pathways have been identified in different GBM patients (51). Another recurrent mutation is the one occurring in the isocitrate dehydrogenase 1 gene (IDH1). This mutation is much more frequent in LGG and secondary GBM (GBM arising from LGG) than in primary GBM, and it is associated with an increased survival (52, 53). Interestingly, IDH1 mutations are associated with the existence of a glioma-CpG island hypermethylation phenotype (G-CIMP tumors), which also correlates with a significantly improved outcome (54). Thus, the study of epigenetics of GBM and the consequence of its mutations is also relevant. Among all epigenetic phenomena, genomic imprinting could be particularly important in GBM since several imprinted genes function as cellular mitogens or tumor suppressors, and misexpression of some of these imprinted genes has been postulated in human GBM (55). For example, repression of the tumor suppressor *CDKN1C* (p57^KIP2^), a maternally expressed gene, or overexpression of an oncogene, such as the paternally-expressed imprinted gene *IGF2*, increases the chance of developing the malignant process (21–23, 56). Precisely, upregulation of *IGF2* as a result of a LOI has been associated with several cancers due to over-proliferation effects (57, 58). Also, the maternally expressed *H19* is overexpressed in GBM samples compared to healthy brains, and its role as an oncogenic lncRNA through inhibition of β–*catenin* expression is clearly recognized (59). Low expression of the maternally expressed gene *MEG3* significantly correlates with short survival in GBM patients, and *in vitro* restoration of *MEG3* impairs tumorigenic abilities of GBM cells (60). Moreover, epigenetic silencing of the paternally expressed gene *PEG3* was confirmed in GBM (61). *Contactin 3* (*CNTN3*), another imprinted gene, has been postulated as a biomarker that predicts overall survival in GBM patients (62). Similarly, expression of the paternally expressed gene *DLK1* is higher in GBM cells than in normal brain thus increasing their proliferation and migration capabilities (63). Therefore, an important role of genomic imprinting in human GBM is starting to also be elucidated.

**Figure 2:**
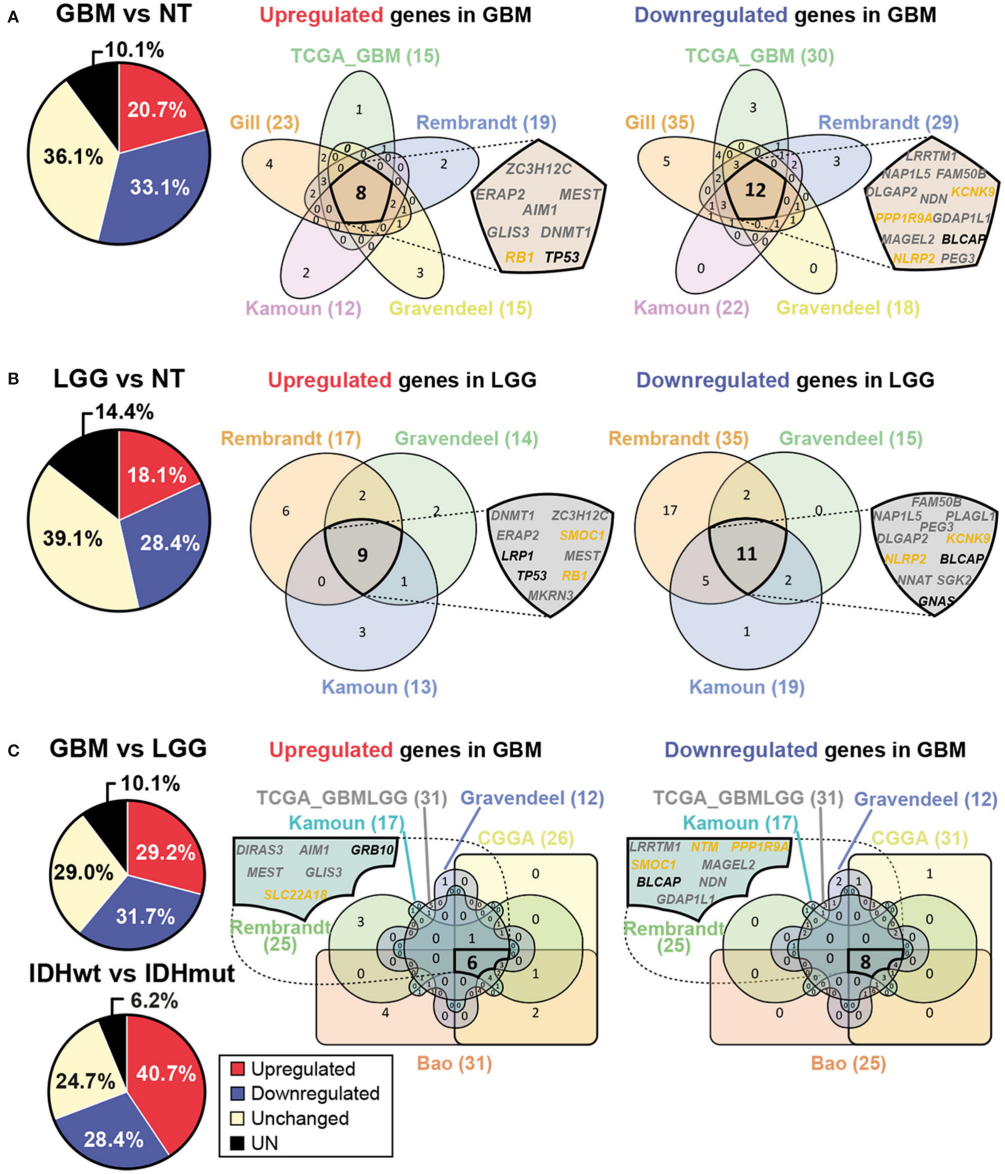
Expression of imprinted genes is altered in human GBM. **(A)** Pie chart representing average percentages of upregulated (red) and downregulated (blue) genes in GBM when compared with non-tumor (NT) samples and obtained with different human GlioVis datasets. Non-significant gene expression is also included (yellow). The average percentages of genes which data are not available are shown in black (UN, left panel). Venn diagrams represent imprinted genes which are upregulated or downregulated in GBM when compared with NT samples. Each dataset used is represented (right panel). Intersection of all sets shows genes which expression pattern is coincident in every analyzed dataset. Maternally expressed genes are indicated in yellow whereas paternally expressed genes are indicated in gray. Genes with unknown specific-parental expression are in black. **(B)** Pie chart representing average percentages of imprinted genes which are upregulated (red) or downregulated (blue) in low grade glioma (LGG) compared to NT samples (left panel). Venn diagrams representing imprinted genes which are differentially expressed between LGG and NT samples (right panel). Intersection of all sets represents genes which expression pattern is coincident in every analyzed dataset. **(C)** Pie charts representing average percentages of imprinted genes which are upregulated (red) or downregulated (blue) in GBM compared to LGG samples and in IDHwt compared to IDHmut LGG samples. Venn diagrams representing imprinted genes which are differentially expressed between GBM and LGG samples (right panel). Intersection of all sets represents genes which expression pattern is coincident in every analyzed dataset. GlioVis datasets used are Bao, CGGA, Gill, TCGA_GBM, TCGA_GBMLGG, Rembrandt, Gravendeel and Kamoun.

In order to corroborate the potential relevance of genomic imprinting in human GBM, we searched for imprinted genes expression in different tumor and non-tumor samples using the GlioVis database (64). Eight datasets were chosen, five of them containing RNAseq data: Bao (65), CGGA (66), Gill (67), TCGA_GBM (68), and TCGA_GBMLGG (69); and the other three containing microarray data: Rembrandt (70), Gravendeel (71) and Kamoun (72). Expression analysis was executed comparing non-tumor (NT), low grade glioma (LGG, grade II-III gliomas), and GBM (grade IV gliomas) human samples. Using these datasets, analysis of the expression of 81 imprinted genes was performed in three different comparisons: GBM and NT samples (GBM vs. NT), LGG and NT samples (LGG vs. NT) and GBM and LGG samples (GBM vs. LGG). Different numbers of datasets were used in each case: five datasets for GBM vs. NT comparison (Gill, TCGA_GBM, Rembrandt, Gravendeel and Kamoun); three datasets for LGG vs. NT comparison (Rembrandt, Gravendeel and Kamoun); and six datasets for GBM vs. LGG (Bao, CGGA, TCGA_GBMLGG, Rembrandt, Gravendeel and Kamoun). The results show that a high number of imprinted genes alter their expression levels in all comparisons. For example, 53.8% of imprinted genes resulted differentially expressed in GBM compared to NT samples (Figure 2A), 46.5% in LGG compared to NT samples (Figure 2B) and 60.9% in GBM compared to LGG samples (Figure 2C). These data support the hypothesis of genomic imprinting having a relevant role in glioma development and progression. Additionally, we have performed a similar analysis comparing the expression of imprinted genes in IDHwt and IDHmut LGG samples using TCGA_GBMLGG database. This study shows that 69.1% of imprinted genes are differentially expressed in IDHwt and IDHmut samples (Figure 2C), suggesting that imprinted genes could also be important for patient prognosis.

Expression patterns in every dataset were analyzed using Venn diagrams (73) and three lists of imprinted genes were obtained from the analysis, one per comparison, each of them containing upregulated and downregulated genes in different samples (Figure 2). A list of 20 differentially expressed genes between GBM and NT samples was obtained from the analysis, 8 of them upregulated (*ZC3H12C, ERAP2, MEST, AIM1, GLIS3, DNMT1, RB1*, and *TP53*) and the other 12 downregulated (*LRRTM1, NAP1L5, FAM50B, DLGAP2, NDN, KCNK9, PPP1R9A, GDAP1L1, MAGEL2, BLCAP, NLRP2*, and *PEG3*) in GBM (Figure 2A). Another list of 20 genes with different expression levels was obtained when comparing LGG and NT samples, 9 of them upregulated (*DNMT1, ZC3H12C, ERAP2, SMOC1, LRP1, MEST, TP53, RB1* y *MKRN3*) and the other 11 genes downregulated (*FAM50B, NAP1L5, PLAGL1, PEG3, DLGAP2, KCNK9, NLRP2, BLCAP, NNAT, SGK2* y *GNAS*) in LGG (Figure 2B). A third list of 14 differentially expressed genes was obtained when comparing both types of tumor samples (GBM and LGG), being 6 of them upregulated (*DIRAS3, AIM1, GRB10, MEST, GLIS3* y *SLC22A18*) and the other 8 downregulated (*LRRTM1, NTM, PPP1R9A, SMOC1, MAGEL2, BLCAP, NDN* and *GDAP1L1*) in GBM (Figure 2C). This analysis reveals potential candidates for future research on the role of concrete imprinted genes and gene dosage control in GBM formation.

## Aberrations of Genomic Imprinting in NSCs and GBM Formation

Due to its similarities with astrocytes, GBM is considered an astrocytoma (45, 46). However, the cell of origin of GBM is not completely understood. Several studies have described the presence of a cell population with stem cell characteristics within the tumors, the glioma stem cells (GSCs), which have GBM potential and are responsible for the origin of the tumors (74–77). These cells can give rise to new tumors by themselves and are thought to be responsible for the resistance to treatment and the high risk of recurrence in this kind of tumor (78). GSCs express stem cell markers, sharing some features with NSCs, such as the expression of some surface antigens and the activation of some signaling pathways (79). In addition, both cell types exhibit a similar proliferation rate, a similar transcriptome and are closely associated to blood vessels (80, 81). Although some authors have demonstrated that differentiated cell types can be reprogrammed and form GBM when bearing some specific-gene mutations (82–84), NSCs have also been proposed to be the cell of origin of GSCs (76, 85, 86). Indeed, some authors have described that susceptibility to malignant transformation of NSCs decreases with the increase of lineage restriction in the brain, suggesting a GBM hierarchy in which NSCs are the most common cell-of-origin and differentiated cell types are less susceptible to tumorigenesis (87).

As we mentioned before, imprinted genes are defined by their monoallelic expression with implications in development and placentation, but also in metabolism of the adult organism (11, 12). These characteristics make these genes extremely susceptible to mutations. LOI most likely precedes tumor formation and several studies suggest this to occur originally in stem cell populations, leading to their transformation (23, 57). This theory posits that epigenetic modifications such as LOI take place in stem cells and this is supported by the presence of non-malignant cells around the tumor with LOI events (23). Indeed, an increase of the stem cell pools due to LOI (for example with high levels of *IGF2*) could favor the accumulation of mutations, creating a suitable context for transformation (21, 57). Thus, genomic imprinting seems to play an important role in converting stem cells into cancer stem cells, although very little is known about how aberrations of genomic imprinting might participate specifically in the malignant transformation of NSCs. It has been recently described that the imprinted lncRNA *MEG3* acts as a tumor-suppressor gene in GSCs, inhibiting cell growth, migration and colony-forming abilities of GSCs *in vitro* (60). Moreover, the imprinted gene *DLK1*, essential for the maintenance of NSCs in the murine adult SVZ (42, 88), increases its expression in human glioma and promotes proliferation of GBM cell lines (60, 63). Nonetheless, the molecular mechanisms governing the tumor suppressing or promoting activities of these genes and other imprinted genes in GBM remain elusive.

**Figure 3:**
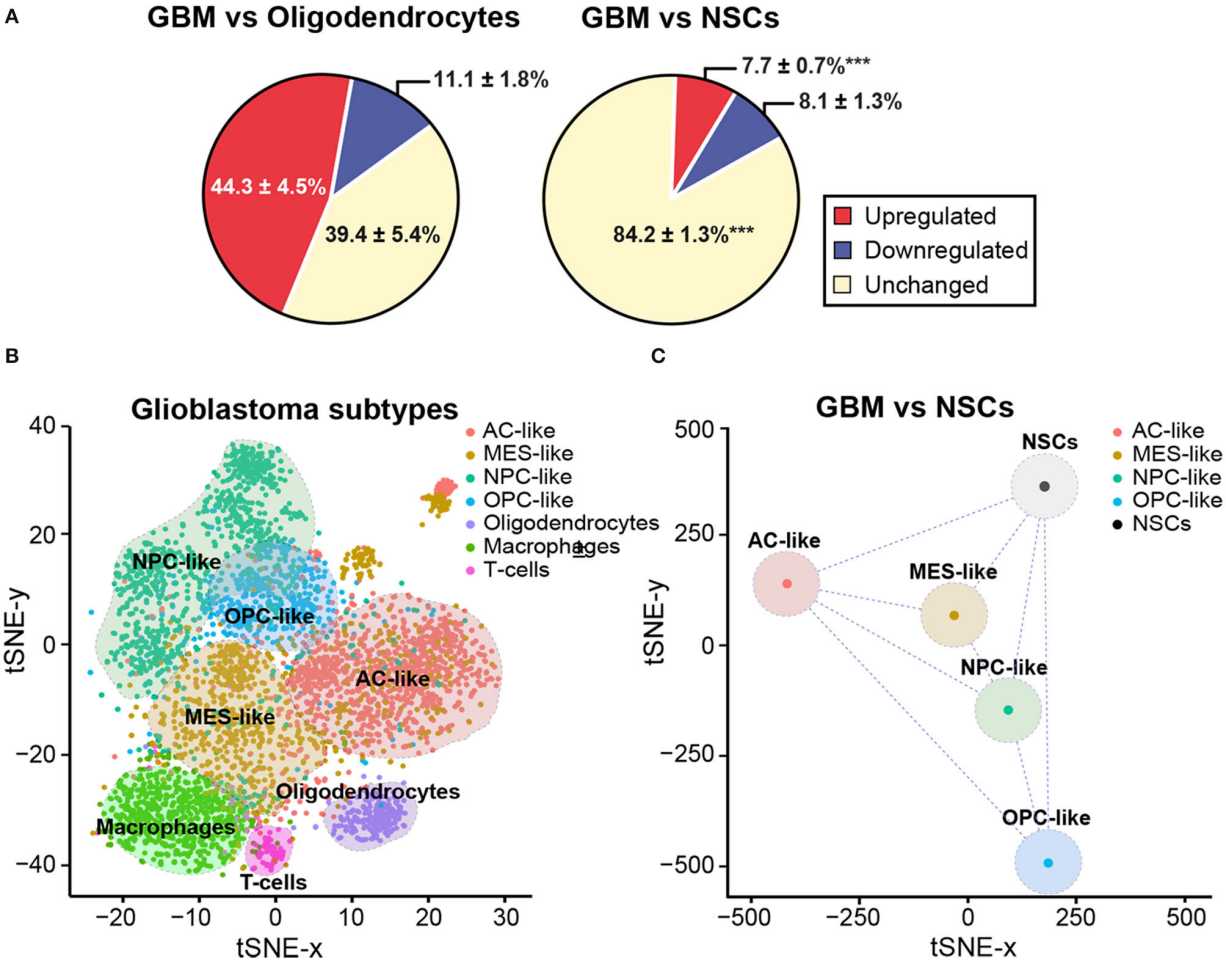
NSCs share imprinting gene expression profile with GBM cells. **(A)** Pie chart representing average percentages of upregulated (red) and downregulated (blue) imprinted genes in GBM when compared with oligodendrocytes (left panel) or NSCs (right panel). Percentage of imprinted genes that do not change their expression levels is also included (yellow). A statistical analysis was performed to determine the changes in the percentage of downregulated, upregulated or unchanged genes in NSCs and oligodendrocytes when compared to GBM. Mean percentage and s.e.m are indicated. *P*-values: ***p < 0.001. **(B)** tSNE dimensional reduction plot of single-cell RNAseq data from Neftel et al. (88) (downloaded from GSE131928) showing that the four GBM cell state subtypes and the three non-malignant cell types form discrete clusters based on the expression of 222 imprinted genes. AC-like (astrocytic-like), MES-like (mesenchymal-like), NPC-like (neural progenitor-like) and OPC-like (oligodendroglial progenitor-like). Non-tumoural cells: oligodendrocytes, macrophages and T-cells. Assignment of cell state names to individual GBM cells was based on the reanalysis of the two-dimensional hierarchical representation of cellular states from Neftel et al. (88). From each of the four quadrants, cells that displayed relative meta-module scores > 1 were selected and named according to their corresponding cellular state, as defined in the figure. In total, 2,528 GBM cells and 1,014 non-malignant cells were used to generate the tSNE plot. **(C)** The tSNE dimensional reduction plot of GBM cells was repeated after converting the scRNAseq data of tumoural states into pseudo-bulk RNAseq data and incorporating to the input expression matrix the four biological replicates of the bulk RNAseq datasets for NSCs from Donega et al. (89) (downloaded from GSE130752).

In order to further elucidate the potential regulation of genomic imprinting during malignant transformation of NSCs, we performed an analysis of single-cell RNA sequencing data, which had been previously generated from 28 human GBM samples (88), and compared it with non-malignant oligodendrocytes and adult human NSCs (89). Of the 222 imprinted genes analyzed, 92 showed significant expression in oligodendrocytes and 68 were expressed in human NSCs. Interestingly, more than 70% of these genes were altered in GBM when compared to non-malignant oligodendrocytes (Figure 3A), whereas only 16% of genes were altered when compared to human NSCs (Figure 3A). This suggests that the transcriptomes of NSCs are more closely related to those of tumor cells than to non-malignant cells.

It has been described that malignant cells in GBM exist in four main cellular states that recapitulate distinct neural cell types within the tumors: oligodendrocyte-progenitor-like (OPC-like), astrocyte-like (AC-like), mesenchymal-like (MES-like) and neural-progenitor-like (NPC-like) states (88). Importantly, plasticity between states and the potential for a single cell to generate all four states have been shown. Based on the same single-cell RNAseq datasets, we performed a tSNE dimensional reduction analysis taking into account only the molecular profiles of the 222 imprinted genes present in the expression matrix. On top of the tSNE plot, cells were color-coded according to their assignment as each of the four tumoral states (88). Based on the expression of imprinted genes only, cells appeared as visually distinctive groups that nicely matched either their cell states in case of GBM cells, or their cell type in case of non-malignant cells (Figure 3B). Non-malignant cells, which highly expressed previously described markers of specific cell types such as oligodendrocytes, macrophages or T cells (88), formed three discrete groups at the bottom of the plot clearly separated from GBM cells (Figure 3B). Aiming to compare the single-cell transcriptomes of GBM cells and bulk RNA-seq datasets previously generated of NSCs (89), we averaged the single-cell datasets to convert them into comparable pseudo-bulk datasets and repeated the dimensional reduction analysis. Interestingly, the resulting plot indicated that the distance in the two-dimensional plane was not higher between NSCs and GBM states than among the four tumoral subtypes (Figure 3C). Our analysis overall indicates that imprinted gene expression programs might have biological significance in tumor identity, thus being of potential value for diagnosis and GBM treatment.

## Concluding Remarks

Genomic imprinting is an epigenetic phenomenon consisting in the expression of imprinted genes only by one allele depending on its parental origin. This process is susceptible to alterations that not only can cause some human syndromes but are also involved in cancer development. Indeed, some imprinted genes act as oncogenes or tumor suppressor genes and have been involved in malignant transformation. In GBM, which is the most frequent and malignant primary brain tumor in humans, the misexpression of some concrete imprinted genes has been previously described. In this review, we show the results of an expression data analysis performed in GBM and non-tumor samples, confirming that an extensive alteration in the expression of imprinted genes does exist in GBM. Although the cell-of-origin of GBM has not been completely elucidated yet, NSCs seem to be good candidates as they share multiple features with GBM cells. There is emerging evidence pointing out that NSCs could undergo malignant transformation and give rise to GBM, and that genomic imprinting could be important in this process. In contrast to other non-malignant cells, adult NSCs from the human SVZ cannot be distinguished from GBM cells based on imprinted gene expression data, supporting the hypothesis that NSCs are the cells-of-origin of GBM. Taken together, all these data reveal genomic imprinting as an important epigenetic mechanism in GBM origin and development, and thus make aberrations of imprinting a potentially valuable tool for both diagnosis and cancer treatment. However, the causal relationship between aberrations of imprinting and GBM formation has not been resolved yet and needs to be studied further in the future.

## Author Contributions

AL-U and EJ-V performed the GlioVis analysis. AJ-P performed the RNAseq data analysis. SF initiated, designed, and wrote the manuscript. All authors contributed to data analysis, discussion, and writing of the paper.

## Conflict of Interest

The authors declare that the research was conducted in the absence of any commercial or financial relationships that could be construed as a potential conflict of interest.

## References

[1] Bartolomei MS. Genomic imprinting: employing and avoiding epigenetic processes. Genes Dev. (2009) 23:2124–33. 10.1101/gad.1841409 19759261 PMC2751984

[2] Ferguson-Smith AC. Genomic imprinting: the emergence of an epigenetic paradigm. Nat Rev Genet. (2011) 12:565–75. 10.1038/nrg3032 21765458

[3] Peters J. The role of genomic imprinting in biology and disease: an expanding view. Nat Rev Genet. (2014) 15:517–30. 10.1038/nrg3766 24958438

[4] Barlow DP Bartolomei MS. Genomic imprinting in mammals. Cold Spring Harb Perspect Biol. (2014) 6:a018382. 10.1101/cshperspect.a018382 24492710 PMC3941233

[5] Ubeda F. Evolution of genomic imprinting with biparental care: implications for Prader-Willi and Angelman syndromes. PLoS Biol. (2008) 6:e208. 10.1371/journal.pbio.0060208 18752349 PMC2525684

[6] da Rocha ST Ferguson-Smith AC. Genomic imprinting. Curr Biol. (2004) 14:R646–9. 10.1016/j.cub.2004.08.007 15324678

[7] Kono T Obata Y Wu Q Niwa K Ono Y Yamamoto Y. Birth of parthenogenetic mice that can develop to adulthood. Nature. (2004) 428:860–4. 10.1038/nature02402 15103378

[8] Li X Li MJ Yang Y Bai Y. Effects of reprogramming on genomic imprinting and the application of pluripotent stem cells. Stem Cell Res. (2019) 41:101655. 10.1016/j.scr.2019.101655 31734645

[9] Monk D Mackay DJG Eggermann T Maher ER Riccio A. Genomic imprinting disorders: lessons on how genome, epigenome and environment interact. Nat Rev Genet. (2019) 20:235–48. 10.1038/s41576-018-0092-0 30647469

[10] Tucci V Isles AR Kelsey G Ferguson-Smith AC Group EI. Genomic imprinting and physiological processes in mammals. Cell. (2019) 176:952–65. 10.1016/j.cell.2019.01.043 30794780

[11] Kim J Bretz CL Lee S. Epigenetic instability of imprinted genes in human cancers. Nucleic Acids Res. (2015) 43:10689–99. 10.1093/nar/gkv867 26338779 PMC4678850

[12] Morison IM Ramsay JP Spencer HG. A census of mammalian imprinting. Trends Genet. (2005) 21:457–65. 10.1016/j.tig.2005.06.008 15990197

[13] Perrera V Martello G. How does reprogramming to pluripotency affect genomic imprinting? Front Cell Dev Biol. (2019) 7:76. 10.3389/fcell.2019.00076 31143763 PMC6521591

[14] MacDonald WA Mann MR. Epigenetic regulation of genomic imprinting from germ line to preimplantation. Mol Reprod Dev. (2014) 81:126–40. 10.1002/mrd.22220 23893518

[15] Godini R Karami K Fallahi H. Genome imprinting in stem cells: a mini-review. Gene Expr Patterns. (2019) 34:119063. 10.1016/j.gep.2019.119063 31279979

[16] Spahn L Barlow DP. An ICE pattern crystallizes. Nat Genet. (2003) 35:11–2. 10.1038/ng0903-11 12947402 PMC2847178

[17] Reik W Walter J. Genomic imprinting: parental influence on the genome. Nat Rev Genet. (2001) 2:21–32. 10.1038/35047554 11253064

[18] Hajkova P Ancelin K Waldmann T Lacoste N Lange UC Cesari F. Chromatin dynamics during epigenetic reprogramming in the mouse germ line. Nature. (2008) 452:877–81. 10.1038/nature06714 18354397 PMC3847605

[19] Lucifero D Mann MR Bartolomei MS Trasler JM. Gene-specific timing and epigenetic memory in oocyte imprinting. Hum Mol Genet. (2004) 13:839–49. 10.1093/hmg/ddh104 14998934

[20] Cassidy SB Schwartz S. Prader-Willi and Angelman syndromes. Disorders of genomic imprinting. Medicine. (1998) 77:140–51. 10.1097/00005792-199803000-00005 9556704

[21] Feinberg AP. An epigenetic approach to cancer etiology. Cancer J. (2007) 13:70–4. 10.1097/PPO.0b013e31803c6e3b 17464249

[22] Holm TM Jackson-Grusby L Brambrink T Yamada Y Rideout WM Jaenisch R. Global loss of imprinting leads to widespread tumorigenesis in adult mice. Cancer Cell. (2005) 8:275–85. 10.1016/j.ccr.2005.09.007 16226703

[23] Jelinic P Shaw P. Loss of imprinting and cancer. J Pathol. (2007) 211:261–8. 10.1002/path.2116 17177177

[24] Kempermann G Song H Gage FH. Neurogenesis in the adult hippocampus. Cold Spring Harb Perspect Biol. (2015) 7:a018812. 10.1101/cshperspect.a018812 26330519 PMC4563705

[25] Obernier K Alvarez-Buylla A. Neural stem cells: origin, heterogeneity and regulation in the adult mammalian brain. Development. (2019) 146:dev156059. 10.1242/dev.156059 30777863 PMC6398449

[26] Lim DA Alvarez-Buylla A. The adult ventricular-subventricular zone (V-SVZ) and olfactory bulb (OB) neurogenesis. Cold Spring Harb Perspect Biol. (2016) 8:a018820. 10.1101/cshperspect.a018820 27048191 PMC4852803

[27] Chaker Z Codega P Doetsch F. A mosaic world: puzzles revealed by adult neural stem cell heterogeneity. Wiley Interdiscip Rev Dev Biol. (2016) 5:640–58. 10.1002/wdev.248 27647730 PMC5113677

[28] Bond AM Ming GL Song H. Adult mammalian neural stem cells and neurogenesis: five decades later. Cell Stem Cell. (2015) 17:385–95. 10.1016/j.stem.2015.09.003 26431181 PMC4683085

[29] Menn B Garcia-Verdugo JM Yaschine C Gonzalez-Perez O Rowitch D Alvarez-Buylla A. Origin of oligodendrocytes in the subventricular zone of the adult brain. J Neurosci. (2006) 26:7907–18. 10.1523/JNEUROSCI.1299-06.2006 16870736 PMC6674207

[30] Sohn J Orosco L Guo F Chung SH Bannerman P Mills Ko E. The subventricular zone continues to generate corpus callosum and rostral migratory stream astroglia in normal adult mice. J Neurosci. (2015) 35:3756–63. 10.1523/JNEUROSCI.3454-14.2015 25740506 PMC6605576

[31] Altmann C Keller S Schmidt MHH. The role of SVZ stem cells in glioblastoma. Cancers. (2019) 11:448. 10.3390/cancers11040448 30934929 PMC6521108

[32] Lagace DC Whitman MC Noonan MA Ables JL DeCarolis NA Arguello AA. Dynamic contribution of nestin-expressing stem cells to adult neurogenesis. J Neurosci. (2007) 27:12623–9. 10.1523/JNEUROSCI.3812-07.2007 18003841 PMC3718551

[33] Merkle FT Tramontin AD Garcia-Verdugo JM Alvarez-Buylla A. Radial glia give rise to adult neural stem cells in the subventricular zone. Proc Natl Acad Sci USA. (2004) 101:17528–32. 10.1073/pnas.0407893101 15574494 PMC536036

[34] Fuentealba LC Rompani SB Parraguez JI Obernier K Romero R Cepko CL. Embryonic origin of postnatal neural stem cells. Cell. (2015) 161:1644–55. 10.1016/j.cell.2015.05.041 26091041 PMC4475276

[35] Furutachi S Matsumoto A Nakayama KI Gotoh Y. p57 controls adult neural stem cell quiescence and modulates the pace of lifelong neurogenesis. EMBO J. (2013) 32:970–81. 10.1038/emboj.2013.50 23481253 PMC3616292

[36] Lozano-Urena A Montalban-Loro R Ferguson-Smith AC Ferron SR. Genomic imprinting and the regulation of postnatal neurogenesis. Brain Plast. (2017) 3:89–98. 10.3233/BPL-160041 29765862 PMC5928554

[37] Rraklli V Södersten E Nyman U Hagey DW Holmberg J. Elevated levels of ZAC1 disrupt neurogenesis and promote rapid in vivo reprogramming. Stem Cell Res. (2016) 16:1–9. 10.1016/j.scr.2015.11.002 26610203

[38] Minamide R Fujiwara K Hasegawa K Yoshikawa K. Antagonistic interplay between necdin and Bmi1 controls proliferation of neural precursor cells in the embryonic mouse neocortex. PLoS ONE. (2014) 9:e84460. 10.1371/journal.pone.0084460 24392139 PMC3879318

[39] Ferrón SR Radford EJ Domingo-Muelas A Kleine I Ramme A Gray D. Differential genomic imprinting regulates paracrine and autocrine roles of IGF2 in mouse adult neurogenesis. Nat Commun. (2015) 6:8265. 10.1038/ncomms9265 26369386 PMC4579569

[40] Lehtinen MK Zappaterra MW Chen X Yang YJ Hill AD Lun M. The cerebrospinal fluid provides a proliferative niche for neural progenitor cells. Neuron. (2011) 69:893–905. 10.1016/j.neuron.2011.01.023 21382550 PMC3085909

[41] Giannoukakis N Deal C Paquette J Goodyer CG Polychronakos C. Parental genomic imprinting of the human IGF2 gene. Nat Genet. (1993) 4:98–101. 10.1038/ng0593-98 8099843

[42] Ferrón SR Charalambous M Radford E McEwen K Wildner H Hind E. Postnatal loss of Dlk1 imprinting in stem cells and niche astrocytes regulates neurogenesis. Nature. (2011) 475:381–5. 10.1038/nature10229 21776083 PMC3160481

[43] McNeill KA. Epidemiology of brain tumors. Neurol Clin. (2016) 34:981–98. 10.1016/j.ncl.2016.06.014 27720005

[44] Louis DN Ohgaki H Wiestler OD Cavenee WK Burger PC Jouvet A. The 2007 WHO classification of tumours of the central nervous system. Acta Neuropathol. (2007) 114:97–109. 10.1007/s00401-007-0243-4 17618441 PMC1929165

[45] Chen R Smith-Cohn M Cohen AL Colman H. Glioma subclassifications and their clinical significance. Neurotherapeutics. (2017) 14:284–97. 10.1007/s13311-017-0519-x 28281173 PMC5398991

[46] Omuro A DeAngelis LM. Glioblastoma and other malignant gliomas: a clinical review. JAMA. (2013) 310:1842–50. 10.1001/jama.2013.280319 24193082

[47] Wirsching HG Galanis E Weller M. Glioblastoma. Handb Clin Neurol. (2016) 134:381–97. 10.1016/B978-0-12-802997-8.00023-2 26948367

[48] Ostrom QT Gittleman H Truitt G Boscia A Kruchko C Barnholtz-Sloan JS. CBTRUS statistical report: primary brain and other central nervous system tumors diagnosed in the United States in 2011-2015. Neuro Oncol. (2018) 20 (suppl_4):iv1–iv86. 10.1093/neuonc/noy131 30445539 PMC6129949

[49] Zahonero C Sanchez-Gomez P. EGFR-dependent mechanisms in glioblastoma: towards a better therapeutic strategy. Cell Mol Life Sci. (2014) 71:3465–88. 10.1007/s00018-014-1608-1 24671641 PMC11113227

[50] Ellis HP McInerney CE Schrimpf D Sahm F Stupnikov A Wadsley M. Clinically actionable insights into initial and matched recurrent glioblastomas to inform novel treatment approaches. J Oncol. (2019) 2019:4878547. 10.1155/2019/4878547 32082376 PMC7012245

[51] Capper D Weissert S Balss J Habel A Meyer J Jager D. Characterization of R132H mutation-specific IDH1 antibody binding in brain tumors. Brain Pathol. (2010) 20:245–54. 10.1111/j.1750-3639.2009.00352.x 19903171 PMC8094636

[52] Parsons DW Jones S Zhang X Lin JC Leary RJ Angenendt P. An integrated genomic analysis of human glioblastoma multiforme. Science. (2008) 321:1807–12. 10.1126/science.1164382 18772396 PMC2820389

[53] Noushmehr H Weisenberger DJ Diefes K Phillips HS Pujara K Berman BP. Identification of a CpG island methylator phenotype that defines a distinct subgroup of glioma. Cancer Cell. (2010) 17:510–22. 10.1016/j.ccr.2010.03.017 20399149 PMC2872684

[54] Uribe-Lewis S Woodfine K Stojic L Murrell A. Molecular mechanisms of genomic imprinting and clinical implications for cancer. Expert Rev Mol Med. (2011) 13:e2. 10.1017/S1462399410001717 21262060

[55] Cui H Cruz-Correa M Giardiello FM Hutcheon DF Kafonek DR Brandenburg S. Loss of IGF2 imprinting: a potential marker of colorectal cancer risk. Science. (2003) 299:1753–5. 10.1126/science.1080902 12637750

[56] Leick MB Shoff CJ Wang EC Congress JL Gallicano GI. Loss of imprinting of IGF2 and the epigenetic progenitor model of cancer. Am J Stem Cells. (2012) 1:59–74. 23671798 PMC3643389

[57] Livingstone C. IGF2 and cancer. Endocr Relat Cancer. (2013) 20:R321–39. 10.1530/ERC-13-0231 24080445

[58] Fazi B Garbo S Toschi N Mangiola A Lombari M Sicari D. The lncRNA H19 positively affects the tumorigenic properties of glioblastoma cells and contributes to NKD1 repression through the recruitment of EZH2 on its promoter. Oncotarget. (2018) 9:15512–25. 10.18632/oncotarget.24496 29643989 PMC5884644

[59] Buccarelli M Lulli V Giuliani A Signore M Martini M D'Alessandris QG. Deregulated expression of the imprinted DLK1-DIO3 region in glioblastoma stem-like cells: tumor suppressor role of lncRNA MEG3. Neuro Oncol. (2020) 22:1771–84. 10.1093/neuonc/noaa127 32459347 PMC7746944

[60] Otsuka S Maegawa S Takamura A Kamitani H Watanabe T Oshimura M. Aberrant promoter methylation and expression of the imprinted PEG3 gene in glioma. Proc Jpn Acad Ser B Phys Biol Sci. (2009) 85:157–65. 10.2183/pjab.85.157 19367087 PMC3524298

[61] Zhu YF Guo YB Zhang HY Yang P Wei DF Zhang TT. Prognostic significance of contactin 3 expression and associated genes in glioblastoma multiforme. Oncol Lett. (2019) 18:1863–71. 10.3892/ol.2019.10482 31423255 PMC6607048

[62] Yin D Xie D Sakajiri S Miller CW Zhu H Popoviciu ML. DLK1: increased expression in gliomas and associated with oncogenic activities. Oncogene. (2006) 25:1852–61. 10.1038/sj.onc.1209219 16288219

[63] Bowman RL Wang Q Carro A Verhaak RG Squatrito M. GlioVis data portal for visualization and analysis of brain tumor expression datasets. Neuro Oncol. (2017) 19:139–41. 10.1093/neuonc/now247 28031383 PMC5193031

[64] Bao ZS Chen HM Yang MY Zhang CB Yu K Ye WL. RNA-seq of 272 gliomas revealed a novel, recurrent PTPRZ1-MET fusion transcript in secondary glioblastomas. Genome Res. (2014) 24:1765–73. 10.1101/gr.165126.113 25135958 PMC4216918

[65] Zhao Z Meng F Wang W Wang Z Zhang C Jiang T. Comprehensive RNA-seq transcriptomic profiling in the malignant progression of gliomas. Sci Data. (2017) 4:170024. 10.1038/sdata.2017.24 28291232 PMC5349247

[66] Gill BJ Pisapia DJ Malone HR Goldstein H Lei L Sonabend A. MRI-localized biopsies reveal subtype-specific differences in molecular and cellular composition at the margins of glioblastoma. Proc Natl Acad Sci USA. (2014) 111:12550–5. 10.1073/pnas.1405839111 25114226 PMC4151734

[67] Network CGAR. Comprehensive genomic characterization defines human glioblastoma genes and core pathways. Nature. (2008) 455:1061–8. 10.1038/nature07385 18772890 PMC2671642

[68] Ceccarelli M Barthel FP Malta TM Sabedot TS Salama SR Murray BA. Molecular profiling reveals biologically discrete subsets and pathways of progression in diffuse glioma. Cell. (2016) 164:550–63. 10.1016/j.cell.2015.12.028 26824661 PMC4754110

[69] Madhavan S Zenklusen JC Kotliarov Y Sahni H Fine HA Buetow K. Rembrandt: helping personalized medicine become a reality through integrative translational research. Mol Cancer Res. (2009) 7:157–67. 10.1158/1541-7786.MCR-08-0435 19208739 PMC2645472

[70] Gravendeel LA Kouwenhoven MC Gevaert O de Rooi JJ Stubbs AP Duijm JE. Intrinsic gene expression profiles of gliomas are a better predictor of survival than histology. Cancer Res. (2009) 69:9065–72. 10.1158/0008-5472.CAN-09-2307 19920198

[71] Kamoun A Idbaih A Dehais C Elarouci N Carpentier C Letouzé E. Integrated multi-omics analysis of oligodendroglial tumours identifies three subgroups of 1p/19q co-deleted gliomas. Nat Commun. (2016) 7:11263. 10.1038/ncomms11263 27090007 PMC4838899

[72] Heberle H Meirelles GV da Silva FR Telles GP Minghim R. InteractiVenn: a web-based tool for the analysis of sets through Venn diagrams. BMC Bioinform. (2015) 16:169. 10.1186/s12859-015-0611-3 25994840 PMC4455604

[73] Auffinger B Spencer D Pytel P Ahmed AU Lesniak MS. The role of glioma stem cells in chemotherapy resistance and glioblastoma multiforme recurrence. Expert Rev Neurother. (2015) 15:741–52. 10.1586/14737175.2015.1051968 26027432 PMC4830899

[74] Bien-Moller S Balz E Herzog S Plantera L Vogelgesang S Weitmann K. Association of glioblastoma multiforme stem cell characteristics, differentiation, and microglia marker genes with patient survival. Stem Cells Int. (2018) 2018:9628289. 10.1155/2018/9628289 29535786 PMC5822829

[75] Lee JH Lee JE Kahng JY Kim SH Park JS Yoon SJ. Human glioblastoma arises from subventricular zone cells with low-level driver mutations. Nature. (2018) 560:243–7. 10.1038/s41586-018-0389-3 30069053

[76] Meyer M Reimand J Lan X Head R Zhu X Kushida M. Single cell-derived clonal analysis of human glioblastoma links functional and genomic heterogeneity. Proc Natl Acad Sci USA. (2015) 112:851–6. 10.1073/pnas.1320611111 25561528 PMC4311802

[77] Lan X Jorg DJ Cavalli FMG Richards LM Nguyen LV Vanner RJ. Fate mapping of human glioblastoma reveals an invariant stem cell hierarchy. Nature. (2017) 549:227–32. 10.1038/nature23666 28854171 PMC5608080

[78] Chesler DA Berger MS Quinones-Hinojosa A. The potential origin of glioblastoma initiating cells. Front Biosci. (2012) 4:190–205. 10.2741/s261 22202053 PMC3635065

[79] Reya T Morrison SJ Clarke MF Weissman IL. Stem cells, cancer, and cancer stem cells. Nature. (2001) 414:105–11. 10.1038/35102167 11689955

[80] Sanai N Alvarez-Buylla A Berger MS. Neural stem cells and the origin of gliomas. N Engl J Med. (2005) 353:811–22. 10.1056/NEJMra043666 16120861

[81] Dai C Celestino JC Okada Y Louis DN Fuller GN Holland EC. PDGF autocrine stimulation dedifferentiates cultured astrocytes and induces oligodendrogliomas and oligoastrocytomas from neural progenitors and astrocytes in vivo. Genes Dev. (2001) 15:1913–25. 10.1101/gad.903001 11485986 PMC312748

[82] Friedmann-Morvinski D Bushong EA Ke E Soda Y Marumoto T Singer O. Dedifferentiation of neurons and astrocytes by oncogenes can induce gliomas in mice. Science. (2012) 338:1080–4. 10.1126/science.1226929 23087000 PMC3595315

[83] Uhrbom L Dai C Celestino JC Rosenblum MK Fuller GN Holland EC. Ink4a-Arf loss cooperates with KRas activation in astrocytes and neural progenitors to generate glioblastomas of various morphologies depending on activated Akt. Cancer Res. (2002) 62:5551–8. 12359767

[84] Alcantara Llaguno SR Wang Z Sun D Chen J Xu J Kim E. Adult lineage-restricted CNS progenitors specify distinct glioblastoma subtypes. Cancer Cell. (2015) 28:429–40. 10.1016/j.ccell.2015.09.007 26461091 PMC4607935

[85] Galvao RP Kasina A McNeill RS Harbin JE Foreman O Verhaak RG. Transformation of quiescent adult oligodendrocyte precursor cells into malignant glioma through a multistep reactivation process. Proc Natl Acad Sci USA. (2014) 111:E4214–23. 10.1073/pnas.1414389111 25246577 PMC4210043

[86] Alcantara Llaguno S Sun D Pedraza AM Vera E Wang Z Burns DK. Cell-of-origin susceptibility to glioblastoma formation declines with neural lineage restriction. Nat Neurosci. (2019) 22:545–55. 10.1038/s41593-018-0333-8 30778149 PMC6594191

[87] Surmacz B Noisa P Risner-Janiczek JR Hui K Ungless M Cui W. DLK1 promotes neurogenesis of human and mouse pluripotent stem cell-derived neural progenitors via modulating notch and BMP signalling. Stem Cell Rev Rep. (2012) 8:459–71. 10.1007/s12015-011-9298-7 21761283

[88] Neftel C Laffy J Filbin MG Hara T Shore ME Rahme GJ. An integrative model of cellular states, plasticity, and genetics for glioblastoma. Cell. (2019) 178:835–49.e21. 10.1016/j.cell.2019.06.024 31327527 PMC6703186

[89] Donega V Burm SM van Strien ME van Bodegraven EJ Paliukhovich I Geut H. Transcriptome and proteome profiling of neural stem cells from the human subventricular zone in Parkinson's disease. Acta Neuropathol Commun. (2019) 7:84. 10.1186/s40478-019-0736-0 31159890 PMC6545684

